# Insights from a multi-institutional registry show duration of endocrine treatment for DCIS impacts second events

**DOI:** 10.1038/s41523-025-00774-3

**Published:** 2025-07-01

**Authors:** Thomas J. O’Keefe, Christina Yau, Emma Iaconetti, Eliza Jeong, Case Brabham, Paul Kim, Joseph McGuire, Ann Griffin, Anne M. Wallace, Laura J. Esserman, Olivier Harismendy, Gillian L. Hirst

**Affiliations:** 1 Department of Surgery, University of California, San Diego, CA USA; 2 Department of Surgery, University of California, San Francisco, CA USA; 3 Moores Cancer Center, Division of Biomedical Informatics, UCSD School of Medicine University of California, San Diego, La Jolla, CA USA; 4 UCSF Helen Diller Family Comprehensive Cancer Center, San Francisco, CA USA

**Keywords:** Surgical oncology, Risk factors, Health care

## Abstract

Ductal carcinoma in situ (DCIS) incidence has risen rapidly with the introduction of screening mammography, yet it is unclear who benefits from both the amount and type of adjuvant treatment (radiation therapy, endocrine therapy) versus what constitutes over-treatment. Our goal was to identify the effects of adjuvant radiation therapy, or endocrine ± radiation therapy versus breast conservation surgery alone in a large multi-center registry of retrospective DCIS cases (*N* = 1916) with median follow up of 7.0 (IQR: 8.43) years. We show that patients with DCIS who took less than 2 years of adjuvant endocrine therapy alone have a similar second event rate as breast conservation surgery. However, patients who took more than 2 years of endocrine therapy show a significantly reduced second event rate, similar to those who received either radiation or combined endocrine + radiation therapy, which was independent of age, tumor size, grade, or period of diagnosis. This highlights the importance of endocrine therapy duration for risk reduction.

## Introduction

Ductal carcinoma in situ (DCIS) has become a common diagnosis, particularly since the introduction of screening mammography, with an estimated 51,000 cases being diagnosed annually in the US in 2022^1^. The percentage of diagnosed breast neoplasms that are DCIS has dramatically increased since the pre-screening era (3% to 20%)^2^. However, it is not clear how much an earlier diagnosis has contributed to the reduction in breast cancer mortality when also considering improved treatments over the same time period, and what constitutes overdiagnosis^3,4^. DCIS presents as a biologically heterogeneous group of breast lesions which vary with respect to histopathologic features and outcomes^5^, yet the standard of care for all DCIS is surgery—either breast-conserving surgery or mastectomy due to the potential for the disease to progress to an invasive lesion^6^. This is followed typically (>80% cases) with adjuvant radiation therapy (RT). The evidence for adjuvant radiation therapy was based on the results of a series of randomized trials in the mid-1980s to early 1990s^7,8^ which showed that radiation therapy reduced subsequent ipsilateral breast cancer by half. The addition of endocrine therapy (ET) to radiation therapy has been shown to have added benefits in terms of reduced risk of both ipsilateral and contralateral events, but there is very limited information on the use of only adjuvant endocrine therapy for DCIS^9–11^. Endocrine therapy includes selective estrogen receptor (ER) modulators such as tamoxifen, which was approved in the US in 1977 for ER+ breast cancer, as well as aromatase inhibitors like anastrozole, which was found in NSABP-35 to be associated with a decreased rate of breast cancer events after breast conservation surgery for DCIS among postmenopausal patients relative to tamoxifen^12^. Retrospective evidence suggests that between 14 and 53% of unresected DCIS lesions will not progress to invasive disease^13,14^, and it has been demonstrated that endocrine therapy reduces the risk of invasive disease in the absence of any surgery in some DCIS^15^, lending support for active surveillance and ET in selected patients.

There is a need for improved algorithms for stratifying risk when considering treatment options for patients diagnosed with DCIS to reduce the potential harms of over-treatment. Currently, NCCN guidelines^16^ recommend either breast conservation surgery with adjuvant radiation therapy or mastectomy for patients with DCIS, as well as sentinel lymph node biopsy for patients undergoing mastectomy, and for patients undergoing breast conservation surgery with high preoperative suspicion for invasive disease. Endocrine therapy is recommended for risk-reduction therapy for patients with ER+ disease. This includes 20 mg/day tamoxifen, for up to 5 years based on data in invasive breast cancer, and for postmenopausal patients, an aromatase inhibitor which has been shown to be of particular benefit among patients less than 60 years old, and those with high risk of thromboembolism. In the event of a recurrence, the treatment plan, including the management of endocrine therapy if it has not yet been completed, must be reassessed. Additionally, there is a growing interest in the development of scoring systems using data from molecular, pathologic and imaging biomarkers to accomplish this. Two such scoring systems have been described to help guide treatment decisions^17,18^ but lack widespread utilization.

One limiting factor is that few large databases exist for DCIS that contain adequate and consistent longitudinal data regarding clinicopathologic details and the outcomes of the patients studied. Another issue is that treatment specifics such as duration of endocrine therapy, which is known to reduce recurrence (ipsilateral and contralateral) in invasive breast cancer^19^ has not been characterized in national registries or institutional studies of DCIS to date. It is quite likely that variability in the duration of endocrine therapy had an impact on the conclusions drawn from historical trials^20^ which has influenced DCIS treatment practice to date. The Vermont DCIS registry^11^ described by Sprague and colleagues is one of the few studies that have examined the impact on second events of just adjuvant endocrine therapy by itself following breast conservation surgery. For these reasons we wanted to conduct a deeper analysis of DCIS treatment and examine specifically the impact of endocrine therapy duration on outcomes. For this purpose, we constructed a database for a large cohort of patients treated for DCIS at the University of California, San Francisco (UCSF) and San Diego (UCSD). This is tied into an ongoing tissue collection for downstream molecular and pathologic analysis. Cancer registry data were confirmed by chart review and supplemented with durations of endocrine therapy for our final analysis cohort. We assessed time-varying risk of recurrence, which included in situ and invasive ipsilateral and contralateral second breast cancer events, by treatment type.

## Results

### Treatment Cohorts

**Fig. 1:**
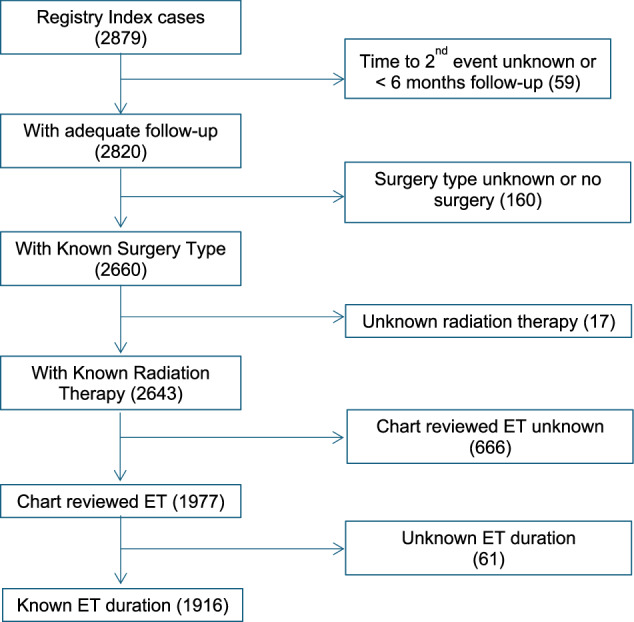
CONSORT Diagram. Only patients with known follow-up of at least six months and known treatment variables including endocrine therapy duration were included. ET endocrine therapy.

2879 index DCIS cases were initially identified from the UCSF and UCSD cancer registries (Fig. 1) covering the period from 1985 to 2017 using our selection criteria. Through subsequent chart review, we excluded patients with missing data regarding surgical type, use of adjuvant radiation or endocrine therapy, duration of endocrine therapy, or patients who developed a second breast cancer event within 6 months of their initial DCIS or had fewer than 6 months of follow-up. Among the 1916 patients who met these criteria, median age at diagnosis was 55 years, and median follow-up time was 7.0 (IQR: 8.43) years. 539 (28%) patients in our cohort had a mastectomy with or without adjuvant endocrine therapy. Among patients who underwent breast conservation surgery, 401 (21%) did not receive any adjuvant treatment (Table 1), 572 (30%) received adjuvant radiation therapy only, 152 (8%) received adjuvant endocrine therapy only, and 252 (13%) received both adjuvant endocrine and radiation therapy.

**Table 1:** Patient Characteristics

	Overall (*N* = 1916)	BCS (*N* = 401)	BCS + RT (*N* = 572)	BCS + ET (*N* = 152)	BCS + ET + RT (*N* = 252)	Mastectomy ± ET (*N* = 539)
Age						
Median (Range)	55 (19–– 95)	59 (30–95)	59 (34–84)	55.5 (19–83)	54.5 (28–78)	49 (24–81)
<50	637 (33.2%)	102 (25.4%)	123 (21.5%)	48 (31.6%)	79 (31.3%)	285 (52.9%)
≥50	1279 (66.8%)	299 (74.6%)	449 (78.5%)	104 (68.4%)	173 (68.7%)	254 (47.1%)
Period of index diagnosis						
1985–1993	53 (2.8%)	17 (4.2%)	6 (1%)	1 (0.7%)	1 (0.4%)	28 (5.2%)
1994–2001	279 (14.6%)	92 (22.9%)	56 (9.8%)	16 (10.5%)	18 (7.1%)	97 (18%)
2002–2009	704 (36.7%)	136 (33.9%)	204 (35.7%)	68 (44.7%)	106 (42.1%)	190 (35.3%)
2010–2017	880 (45.9%)	156 (38.9%)	306 (53.5%)	67 (44.1%)	127 (50.4%)	224 (41.6%)
Size						
≤2 cm	963 (67.7%)	228 (80%)	332 (73.6%)	86 (84.3%)	130 (67.4%)	187 (47.7%)
>2 cm	460 (32.3%)	57 (20%)	119 (26.4%)	16 (15.7%)	63 (32.6%)	205 (52.3%)
Unknown	493	116	121	50	59	147
Grade						
Low	113 (6.8%)	31 (9.3%)	29 (5.8%)	18 (13.6%)	12 (5.1%)	23 (4.9%)
Intermediate	694 (41.8%)	162 (48.8%)	187 (37.6%)	71 (53.8%)	108 (46%)	166 (35.7%)
High	855 (51.4%)	139 (41.9%)	282 (56.6%)	43 (32.6%)	115 (48.9%)	276 (59.4%)
Unknown	254	69	74	20	17	74
Comedonecrosis						
No	534 (48.5%)	127 (62.3%)	181 (50.1%)	44 (65.7%)	67 (42.4%)	115 (36.9%)
Yes	568 (51.5%)	77 (37.7%)	180 (49.9%)	23 (34.3%)	91 (57.6%)	197 (63.1%)
Unknown	814	197	211	85	94	227
ER Status						
Positive	1080 (83.5%)	192 (86.1%)	335 (79.2%)	100 (98%)	196 (95.6%)	257 (75.6%)
Negative	213 (16.5%)	31 (13.9%)	88 (20.8%)	2 (2%)	9 (4.4%)	83 (24.4%)
Unknown	623	178	149	50	47	199
Endocrine therapy duration						
≤2	155 (8.1%)	0	0	58 (38.2%)	64 (25.4%)	33 (6.1%)
>2	318 (16.6%)	0	0	94 (61.8%)	188 (74.6%)	36 (6.7%)
No ET	1443 (75.3%)	401 (100%)	572 (100%)	0	0	470 (87.2%)
Institution						
UCSD	996 (52%)	160 (40%)	363 (63%)	56 (37%)	170 (67%)	247 (46%)
UCSF	920 (48%)	241 (60%)	209 (37%)	96 (63%)	82 (33%)	292 (54%)
Follow-up						
Time to last follow-up, Median (Range), yrs	8.2 (0.5–32.8)	9.2 (0.6–32.8)	6.7 (0.5–29.6)	9.4 (0.6–24.6)	7.6 (0.5–28.6)	9.0 (0.5–31.1)
Number of second events	194	75	41	16	15	47

*BCS* breast conservation surgery, *RT* radiation therapy, *ET* endocrine therapy, *ER* estrogen receptor, *yrs* years.

Patients diagnosed after 2001 were more likely to receive adjuvant endocrine (*p* = 0.0018) or radiation (*p* < 0.0001) therapy than those diagnosed before 2001 (Table 1). Patients who were younger (median age 49, *p* < 0.0001), had larger lesion size (*p* < 0.0001), had higher grade disease (*p* = 0.0002), and ER negative disease (*p* < 0.0001) were more likely to undergo mastectomy rather than those who had breast conservation surgery, with or without adjuvant therapy. Among patients undergoing breast conservation surgery, patients with larger lesion size (*p* < 0.0001) and higher grade disease (*p* < 0.0009) were more likely to receive adjuvant radiation therapy.

Among the 823 patients with ER+ disease who underwent breast conservation therapy, 296 (36.0%) received adjuvant endocrine therapy as a part of their treatment regimen, 335 (40.7%) received adjuvant radiotherapy only, and 192 (23.3%) received no adjuvant therapy. Among the 213 patients with confirmed ER- DCIS, 11 (5.2%) received adjuvant endocrine therapy as part of their treatment. A large number of cases (*N* = 623, 33%), particularly those diagnosed in the earlier years of our study, did not have receptor data available (Table 1).

More than half of patients (70%) who received breast conservation surgery with adjuvant endocrine therapy alone or with radiation therapy continued their treatment for more than 2 years. The majority (87%) of patients receiving mastectomy did not receive any form of endocrine therapy, with 257 of the 539 (76%) of them having ER+ disease.

### Cumulative incidence of second events by treatment type

1916 patients received surgery (either breast conservation surgery or mastectomy) with or without endocrine or radiation therapy, with median follow-up of 7.0 (IQR: 8.43) years. 194 patients (10%) experienced a second breast cancer event (Table 1) with the majority of events occurring in the first 6 years (Supplemental Table 1). Interestingly, none of the patient characteristics captured (period of diagnosis, lesion size, grade, presence of comedonecrosis, and ER status), except age, was associated with risk of a second event. The risk of a second event was lower in the first 7.5 years and higher after 7.5 years in patients ≥50 when compared to those <50, reflecting differences in the timing of risk between younger and older patients (Supplemental Table 2).

**Fig. 2:**
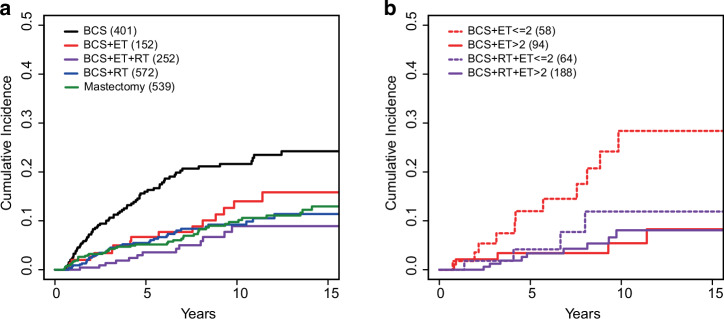
Cumulative Incidence of Second Events by Treatment Type. **a** Cumulative incidence curves of second events by treatment type: BCS (black), BCS + ET (red), BCS + ET + RT (purple), BCS + RT (blue), Mastectomy (green). **b** Cumulative incidence curves for ET treated patients further stratified by duration of ET: BCS + ET ≤ 2 years (dotted red), BCS + ET > 2 years (solid red), BCS + RT + ET ≤ 2 years (dotted purple), BCS + RT + ET > 2 years (solid purple). BCS= breast conservation surgery, ET endocrine therapy, RT radiation therapy.

Cumulative incidence of second breast cancer events was calculated, and when compared with breast conservation surgery, patients in the other treatment groups had a significantly lower risk of developing any second event up to 15 years. (Fig. 2A and Table 2). This risk reduction remains significant in a Cox multivariate model adjusting for age, size of lesion, grade, period of diagnosis, and institution. (Table 2).

**Table 2:** Association between second events and treatment type

	Univariate			Multivariate^a^			Kaplan Meier Estimates of (any) Second Event Rate		
	*N*	Hazard Ratio (95% CI)	Wald test p	*N*	Hazard Ratio (95% CI)	Wald test p	5 yr	10 yr	15 yr
Treatment type (5-level factor)									
BCS	401	REF		257	REF		16% (12%-20%)	22% (17%-26%)	24% (19%-29%)
BCS + RT	572	0.40 (0.27–0.59)	<0.0001	407	0.34 (0.2–0.56)	<0.0001	5% (3%–7%)	9% (6%–12%)	11% (8%–15%)
BCS + (any)ET	152	0.53 (0.31–0.91)	0.0208	96	0.37 (0.18–0.78)	0.0094	7% (2%–11%)	14% (7%–21%)	16% (8%–23%)
BCS + (any)ET + RT	252	0.27 (0.15–0.5)	<0.0001	186	0.34 (0.17–0.65)	0.0014	4% (1%–6%)	9% (4%–14%)	9% (4%–14%)
Mastectomy	539	0.41 (0.29–0.6)	<0.0001	357	0.32 (0.2–0.53)	<0.0001	5% (3%–7%)	10% (7%–13%)	13% (9%–17%)

^a^Multivariate model adjusting for age, period of diagnosis, tumor size, grade and institution, with time dependent coefficients for age and size.

*CI* confidence interval, *yr* year, *BCS* breast conservation surgery, *REF* reference, *RT* radiation therapy, *ET* endocrine therapy.

Patients who were treated with only breast conservation surgery had the highest estimated number of events by treatment type (16% at 5 years), which increased with time to 24% at 15 years (Table 2). In comparison, the estimated number of second events at 5 years was 5% in patients treated with adjuvant radiation therapy and 4% at 5 years with both adjuvant radiation and endocrine therapy. Similarly, patients treated with breast conservation surgery and endocrine therapy without radiation had an estimated 7% risk of second events at 5 years. Although estimated risks increased over time for all treatment groups, there was still a reduced risk relative to breast conservation surgery alone in both the breast conservation surgery with radiation (11%), breast conservation surgery with radiation and endocrine therapy (9%), as well as breast conservation surgery with endocrine therapy (16%) treatment groups at 15 years.

Strikingly, when patients who received any endocrine therapy were subdivided into those who continued treatment for greater than 2 years and those who did not, significant risk reduction (relative to breast conservation surgery) was only observed among those who received at least 2 years of treatment (Fig. 2B and Table 3). This risk reduction was apparent in both patients who were treated with breast conservation surgery with adjuvant endocrine therapy (HR = 0.15, *p* = 0.008) as well as breast conservation surgery with adjuvant radiation therapy and endocrine therapy (HR = 0.32, *p* = 0.003).

**Table 3:** Association between second events and treatment type stratified by endocrine therapy duration (<2 yr vs. ≥2 yrs)

	Univariate			Multivariate^a^			Kaplan Meier Estimates of (any) Second Event Rate		
	*N*	Hazard Ratio (95% CI)	Wald test p	*N*	Hazard Ratio (95% CI)	Wald test p	5 yr	10 yr	15 yr
Treatment type (7-level factor)									
BCS	401	REF		257	REF		16% (12%−20%)	22% (17%−26%)	24% (19%−29%)
BCS + RT	572	0.40 (0.27−−0.59)	<0.0001	407	0.33 (0.2−0.56)	<0.0001	5% (3%−7%)	9% (6%−12%)	11% (8%−15%)
BCS + ET(≤2)	58	1.00 (0.53−1.88)	0.9961	35	0.75 (0.32−1.75)	0.5039	12% (2%−21%)	28% (11%−42%)	28% (11%−42%)
BCS + ET(>2)	94	0.26 (0.1−0.64)	0.0035	61	0.15 (0.04−0.6)	0.0078	3% (0%−7%)	5% (0%−11%)	8% (0%−16%)
BCS + RT + ET(≤2)	64	0.39 (0.14−1.06)	0.0642	45	0.39 (0.12−1.25)	0.1139	4% (0%−10%)	12% (0%−23%)	12% (0%−23%)
BCS + RT + ET(>2)	188	0.24 (0.12−0.49)	0.0001	141	0.32 (0.15−0.68)	0.0031	3% (0%−6%)	8% (3%−13%)	8% (3%−13%)
Mastectomy	539	0.41 (0.29−0.6)	<0.0001	357	0.32 (0.2−0.53)	<0.0001	5% (3%−7%)	10% (7%−13%)	13% (9%−17%)

^a^Multivariate model adjusting for age, period of diagnosis, tumor size, grade and institution, with time dependent coefficients for age and size.

*CI* confidence interval, *yr* year, *BCS* breast conservation surgery, *REF* reference, *RT* radiation therapy, *ET* endocrine therapy.

The benefit of taking more than 2 years of endocrine therapy was also observed over time, with an estimated 8% risk of second events at 15 years in both patients receiving breast conservation surgery with endocrine therapy (>2 years) and breast conservation surgery with radiation and endocrine therapy (>2 years). In comparison, patients receiving endocrine therapy for less than 2 years duration without radiation had a 28% estimated risk of a second breast cancer event at 15 years. Those receiving endocrine therapy for less than 2 years but received adjuvant radiation had a 12% risk, similar to the estimated risk seen in the breast conservation surgery with radiation treatment group (11%). To address the concern for survivor bias, we excluded patients with less than 2 years of follow-up and/or recurrence within 2 years and saw similar results to the full cohort analysis (Supplemental Table 3).

In addition, we confirmed the benefit of receiving >2 years of endocrine therapy within the subset of patients who received breast conservation surgery without radiation (HR = 0.26 (0.10− 0.64), *p* = 0.004). Among those who received radiation, the further benefit of endocrine therapy was not significant (HR = 0.60 (0.29–1.24), *p* = 0.166), likely due to a smaller number of events among radiation-treated patients (Supplemental Table 4).

In a propensity score-matched analysis, considering treatment as a 7-class variable, all adjuvant therapies and mastectomy were associated with a significantly reduced risk of second breast cancer events, except for breast conservative therapy with adjuvant endocrine therapy taken for a duration of less than 2 years, similar to the unmatched analysis (Supplemental Table 5).

An analysis restricted to ER+ patients was performed in the 1080 known ER+ cases. Supplemental Table 6 shows the univariate Cox model results. Similar to the full cohort analysis, the addition of radiation or endocrine therapy to breast-conserving surgery reduces the risk of second events, and the benefit of endocrine therapy is primarily observed among patients who received at least two years of endocrine therapy.

### Competing risks for type of second events in patients receiving breast conservation surgery

**Fig. 3:**
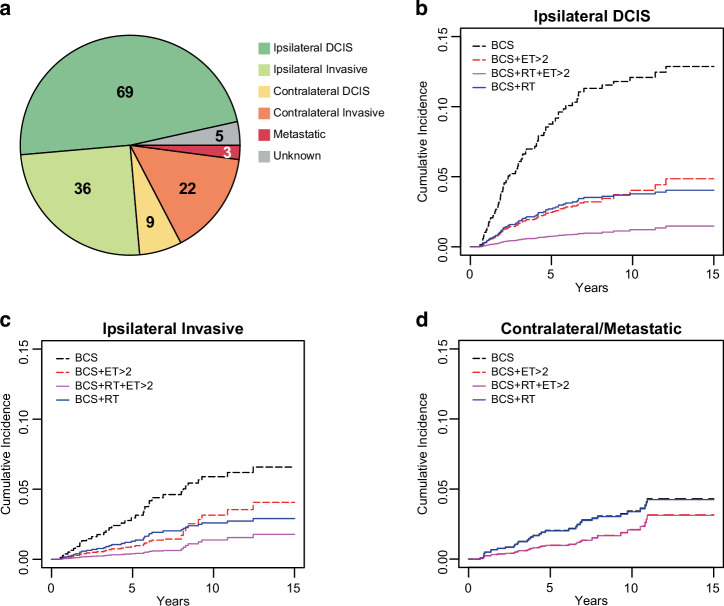
Type of Second Event and Cumulative Incidence by Treatment Type. **a** Distribution of type of second events among patients undergoing BCS. Competing risks model predicted cumulative incidence curves of (**b**) Ipsilateral DCIS, (**c**) Ipsilateral Invasive, and (**d**) Contralateral or Metastatic Second Events by Treatment Type. Patients who received ≤ 2 years of ET were grouped with those who did not receive ET: BCS (alone or with ET ≤ 2 years) (black dashed), BCS + ET > 2 (red dashed), BCS + RT + ET > 2 (purple), BCS + RT (alone or with ET ≤ 2 years) (blue). BCS breast conservation surgery, ET endocrine therapy, RT radiation therapy.

A competing risks model with time-varying coefficients was developed to estimate risk of different second event types among patients undergoing breast conservation surgery as a function of adjuvant endocrine therapy and radiation with censoring at 15 years. For this analysis we removed the 539 mastectomy cases. Median follow-up was 6.5 years (IQR: 7.99). There were 144 events, including 3 metastatic events and 5 unknown events (Fig. 3A). The majority of second events were ipsilateral (105, 73%), with 69 (66%) of them being DCIS. There were 31 contralateral second events (22%), and a majority of them (71%) were invasive, supporting the premise that DCIS is a marker of global risk.

Among all patients, the cumulative incidence of ipsilateral DCIS recurrence was 7%, and the cumulative incidence of ipsilateral invasive and contralateral occurrence were both 4% at 15 years, when considering only second events. In a multivariate competing risks model (Table 4), radiation therapy significantly reduced the risk of both ipsilateral DCIS (HR = 0.29, *p* < 0.0001) and ipsilateral invasive events (HR = 0.42, *p* = 0.01) as expected within the first 7.5 years compared to breast conservation surgery without adjuvant therapy. This risk reduction was not observed between 7.5 and 15 years in our model. Without consideration of duration, any amount of endocrine therapy also significantly reduced the risk of ipsilateral DCIS events within the first 7.5 years (HR = 0.44, *p* = 0.02) (Table 4) and was associated with a non-significant risk reduction for ipsilateral invasive disease (HR = 0.53, *p* = 0.19) and contralateral DCIS (HR = 0.43, *p* = 0.12) in the first 7.5 years. In comparison, radiation therapy by itself was not associated with a risk reduction benefit for contralateral events (HR = 0.97, *p* = 0.93).

**Table 4:** Competing risks analysis of type of second event by treatment type

		Hazard Ratio (CI)	*p*			Hazard Ratio (CI)	*p*
Ipsilateral invasive (Number of events by time period: 0−7.5 yr: 28; 7.5–15 yr: 8)							
RT		0.42 (0.21–0.83)	0.01	RT		0.43 (0.22–0.85)	0.02
(any)ET^a^	0– 7.5 yr	0.53 (0.2–1.38)	0.19	ET > 2 yr^a^	0–7.5 yr	0.31 (0.07–1.27)	0.10
	7.5– 15 yr	1.46 (0.35–6.01)	0.60		7.5– 15 yr	1.30 (0.26–6.35)	0.75
Ipsilateral DCIS (Number of events by time period: 0–7.5 yr: 64; 7.5–15 yr: 5)							
RT		0.29 (0.17–0.48)	<0.0001	RT		0.30 (0.18-0.5)	<0.0001
(any)ET^a^	0–7.5 yr	0.44 (0.23-0.87)	0.02	ET > 2 yr^a^	0–7.5 yr	0.27 (0.1–0.75)	0.01
	7.5– 15 yr	9.73 (1.13–83.77)	0.04		7.5– 15 yr	0.97 (0.11–8.65)	0.98
Contralateral/Metastatic (Number of events by time period: 0–7.5 yr: 26; 7.5–15 yr: 8)							
RT		0.97 (0.49–1.91)	0.93	RT		0.99 (0.5–1.93)	0.97
(any)ET^a^	0–7.5 yr	0.43 (0.15–1.23)	0.12	ET > 2 yr^a^	0–7.5 yr	0.48 (0.14–1.61)	0.23
	7.5– 15 yr	1.39 (0.34–5.78)	0.65		7.5– 15 yr	1.18 (0.25–5.69)	0.83

^a^ET effect modeled using time dependent coefficient.

*CI* confidence interval, *yr* year, *RT* radiation therapy, *ET* endocrine therapy.

Figure 3B–D shows the predicted cumulative incidence curves for ipsilateral DCIS, ipsilateral invasive and contralateral or metastatic events, respectively, by treatment type, where endocrine therapy is defined as patients taking greater than 2 years of endocrine therapy. By stratifying patients undergoing breast conservation surgery with endocrine therapy into those receiving greater than 2 years of endocrine therapy from those receiving less than 2 years, we see a significant further risk reduction (HR = 0.27, *p* = 0.0120) for ipsilateral DCIS second events and a possible further benefit for ipsilateral invasive events (HR = 0.31, *p* = 0.10), within the first 7.5 years only (Table 4). There was also an associated non-significant risk reduction in contralateral disease occurrence (HR = 0.48, *p* = 0.23).

## Discussion

In this study of index DCIS cases at two high-volume academic medical centers, we examined treatment patterns over a 30-year period and found that adjuvant endocrine therapy administered for at least 2 years significantly reduced ipsilateral recurrence of DCIS and non-significantly reduced ipsilateral invasive recurrence as well as contralateral second events. Our findings regarding the impact of endocrine therapy on contralateral second events, which were not observed with the use of adjuvant radiation, are consistent with prior findings by Sprague et al.^11^.

During the past twenty years, the increased utilization of screening mammography has inadvertently led to a rise in the diagnosis and subsequent treatment of DCIS^21^. The standard of care for these lesions includes resection, either mastectomy or breast conservation surgery. When breast conservation surgery is performed, adjuvant radiation therapy is typically administered, and for patients with ER-positive lesions, adjuvant endocrine therapy can be offered. However, this indiscriminate approach to the treatment of a disease as heterogeneous as DCIS likely results in the overtreatment of many patients. The development of approaches to de-escalation of therapy is challenging, in part because of the scarcity of studies on the natural history of untreated DCIS^22–24^. Data from SEER, which must be interpreted with caution, suggest that the risk of developing invasive disease by 10 years for an untreated, low-grade DCIS is 3–4%. For all DCIS lesions, regardless of grade, the risk is around 12%, whether they are surgically resected or not, with a breast cancer-specific survival at 10 years of 98%^13,14,25^.

Importantly, we included the duration of endocrine therapy in our analysis, a variable we had to curate from the electronic medical records, as it is not routinely collected by cancer registries. We found a similar reduction in risk of second breast cancer events to that of adjuvant radiation therapy, when adjuvant endocrine therapy was taken for 2 years or more. We sought to divide patients receiving adjuvant endocrine therapy into two groups with respect to duration. We selected a cutoff of 2 years based on results from early Swedish trials of endocrine therapy, which demonstrated improved long-term outcomes for postmenopausal and premenopausal women with early invasive breast cancer who were treated with adjuvant endocrine therapy for at least 2 years relative to no adjuvant endocrine therapy^26–28^. Additionally, 2 years of therapy was previously considered the standard of care for early invasive breast cancer prior to the discovery that 5 years of therapy was associated with superior outcomes^29^. When we evaluated the effect of endocrine therapy on the type and laterality of second breast cancer events, there was a significant reduction in risk of ipsilateral DCIS as well as a non-significant reduction in ipsilateral invasive disease, with further reduction in risk among patients who received more than 2 years of endocrine therapy. Our results also suggest a reduction in the risk of contralateral events with endocrine therapy, which was not noted among patients receiving radiation therapy without endocrine therapy. Prior studies have also shown a reduction in the risk of contralateral events with the use of adjuvant endocrine therapy^7,9,10^. These benefits of endocrine therapy must be weighed against the known risks associated with the use of ET. There is an increased risk of thromboembolic disease and endometrial cancer with conventional dosing of endocrine therapy^30,31^.

DeCensi and Lazzeroni et al.^32^ recently demonstrated in the Tam01 study that a 5 mg dose of tamoxifen taken daily for 3 years in ADH (atypical ductal hyperplasia), LCIS (Lobular carcinoma in situ), and DCIS patients reduced breast cancer second events by 50% with limited side-effects. Erikkson et al.^33^ found that women undergoing screening had a non-inferior reduction in breast density at a dose as low as 2.5 mg tamoxifen relative to 20 mg, and had a roughly 50% reduction in severe vasomotor symptoms. Further studies would be needed to assess whether lower doses of endocrine therapy for DCIS would be beneficial in the reduction of risk of second breast cancer events and whether a lower dose that is tolerated and taken consistently would improve outcomes relative to ending endocrine therapy if not tolerated at the standard dose.

We opted to focus our analysis on the effect of treatment with respect to the composite outcome of overall second breast cancer event rates, even though radiation and unilateral mastectomy only confer unilateral benefit. We took this approach because we feel it is likely more important to women whether they have any recurrence than whether their recurrence is ipsilateral or contralateral to their index lesion. Particularly given current trends towards identifying safe pathways for de-escalation of treatment for DCIS, we suggest that it is worth reframing the focus of DCIS outcomes to the assessment of any recurrence in an effort to identify patients at highest risk and optimize their management. While reduction in recurrences has not been shown to have an impact on survival outcomes^8^, if the patient’s goal is to reduce the risk of recurrence, then overall recurrence risk rather than ipsilateral vs. contralateral risk represents the most sensible target.

The greatest strength of this study is the supplementation of tumor registry data with curated data for such variables as second events and duration of endocrine therapy. Another strength is the use of two centers for the study—single-institution studies are more prone to institution-specific treatment patterns and biases related to the institution’s patient population. Limitations include the lack of consistent availability of data such as margin status, histopathologic features like comedonecrosis, and, importantly, ER status in some cases. This is important because post-hoc analysis of data from NSABP B-24 demonstrated that endocrine therapy was most effective in risk reduction among patients with ER-positive disease^34^. Since the standard of care involves offering endocrine therapy to patients with ER-positive DCIS, and ER-positive DCIS has a lower risk of recurrence, it is possible that for the full cohort analysis, the observed associated improved outcomes for patients receiving endocrine therapy may have been mediated through its use in patients with ER-positive disease. When we restrict our analysis to just ER+ patients, we still see similar benefits, suggesting that inclusion of the ER- cases is not contributing to the endocrine therapy risk reduction we observe. The endocrine therapy effect on second event risk reduction was not significant among patients who received radiation, likely due to a small number of second events, in spite of a larger number of patients in this group. The association of such features as high-grade and larger lesion size with radiation therapy may result in treatment groups with different aggressiveness of disease, potentially reducing the risk reduction we observe for radiation therapy. However, similar results were observed when we performed a propensity score weighted analysis adjusting for tumor size, grade, and other features. Another significant limitation is the retrospective nature of the study, which precludes the disentanglement of the early discontinuation of endocrine therapy from possibly prognostic biologic factors that may affect tolerance of endocrine therapy, rather than discontinuation resulting purely from patient preference. Treatment patterns also changed over the duration of study, allowing for the possibility that differences in outcomes may have been related to prevalence of each treatment modality over time rather than the treatment modality itself. Finally, margin status represents a source of bias that might favor endocrine therapy. Surgical margins were not routinely required during the early portion of the duration of the study when breast conservation therapy with or without adjuvant radiation would have been standard, and endocrine therapy was not yet part of standard of care for DCIS, in which case local recurrence secondary to inadequate margins may have led to a disproportionate number of ipsilateral recurrences in the breast conservation and radiation therapy only groups.

Many women are understandably confused by their personal risk and the treatment options available to them^35^. Anxiety surrounding this poorly characterized risk likely contributed to the poor recruitment to active surveillance trials for DCIS, like LORIS and LORD, which offered randomization to surveillance and annual mammogram for low-risk DCIS vs. standard of care^36^ and are now being re-designed. Efforts in biomarker development and testing for additional markers such as HER2 in DCIS for risk stratification^18,37–39^, combined with evidence for the use of more tolerable endocrine therapies for both invasive breast cancer and DCIS^40–42^ should help refine treatment approaches. Women with ER + DCIS may be at elevated risk for an invasive cancer event, where the addition of endocrine risk-reducing therapy is effective^43^. The results of our real-world outcomes data analysis support the use of endocrine therapy for risk reduction when tolerated, with additional benefits likely for contralateral risk reduction.

## Methods

This study was approved in concordance with the Declaration of Helsinki by the institutional review boards of UC San Francisco and UC San Diego, utilizing the single UC Reliance mechanism for multi-institutional studies (IRB 16-20550), and was determined minimal risk. As such, patient consent was waived. Patient-identifying information was de-identified and recoded before the data was analyzed and submitted to the database for analysis.

### Patient Selection

Patients both diagnosed with and treated for index DCIS at UCSF and UCSD were identified through the respective cancer center registries. All patients identified were chart reviewed using the electronic medical record system with protocol approval from each center’s institutional review board. Inclusion criteria stipulated patients were at least 18 years of age at the time of diagnosis, underwent unilateral breast conservation surgery or mastectomy for unilateral pure DCIS only, had no other malignancies, breast or otherwise, prior to their DCIS diagnosis and did not have a second event within 6 months of the initial DCIS surgery date. Additionally, to confirm presence or absence of second events, our criteria stipulated that (1) patients undergoing breast conservation surgery have some form of breast imaging at least 6 months after their DCIS surgery, and (2) patients undergoing mastectomy had a documented physical exam at least 6 months after their surgery. Patients were excluded if they had bilateral DCIS, had invasive disease in addition to DCIS, or did not have at least 6 months of follow-up with documentation of appropriate imaging or physical exam findings at that time. Last date of follow-up was based on the most recent clinic note or imaging report. We did not include sex as an eligibility criterion since male DCIS is treated similarly to female DCIS. However, DCIS in men is extremely rare, representing ~9% of about 2700 male breast cancer cases annually, based on the Surveillance, Epidemiology, and End Results (SEER)^44^. 5 cases were included in our analysis cohort.

### Study variables

We collected and chart reviewed: age at diagnosis of index DCIS, type of surgery performed, receipt of radiation therapy (RT), receipt of endocrine therapy (ET), duration of endocrine therapy, lesion laterality, tumor grade, presence or absence of comedonecrosis, and ER status. For each index DCIS, we determined whether there was development of a second breast cancer event, including either a DCIS recurrence or a progression to invasive disease, date of diagnosis, laterality of second event, and most recent negative breast imaging for patients undergoing breast conservation surgery or exam for patients undergoing mastectomy.

### Statistical analysis

Statistical analysis was conducted using R package version 4.2.3.

This study is a retrospective analysis of prospectively collected cancer registry data. A complete case analysis was performed for each analysis conducted, with exclusion of patients with missing data for that analysis. For patients who had a second breast cancer event, the time between their initial DCIS diagnosis and their second event was calculated. For patients who did not have a second breast cancer event, the time from their initial diagnosis to their last follow-up date was calculated. Follow-up time is censored at 15 years. An additional analysis setting the start time at 2 years after diagnosis was also performed.

Each class of clinical variable (age, period of index diagnosis, size, grade, and ER status) was categorized into two or more groups for analysis; and associations with treatment groups (mastectomy vs. breast conservation surgery, radiation vs. no radiation therapy, and endocrine therapy vs. no endocrine therapy) were assessed using the chi-square test. Association between time to second event and clinical covariates, as well as treatment type adjusting for clinical covariates, including age, period of diagnosis, lesion size, and grade, and institution (UCSF vs. UCSD), was assessed using Cox proportional hazard models. Proportional hazard assumptions were tested using Schoenfeld residuals. Where appropriate (for age and size variables), we used time-dependent coefficients (for <7.5 years and ≥7.5 years) to address violations in proportional hazard assumptions. A subset analysis restricted to ER+ patients was also performed. As well, we evaluated endocrine therapy benefit within subsets of patients receiving breast-conserving surgery with and without radiation therapy. In addition, we assessed the effects of radiation and endocrine therapy use on specific types of second events (ipsilateral invasive vs. ipsilateral in situ vs. contralateral) among patients who received breast conservation surgery using competing risks models with time-dependent coefficients where appropriate (for endocrine therapy).

We used propensity score matching to estimate the average treatment effect on the risk of any second breast cancer event in the population, accounting for confounding by the included covariates. Weights were estimated using generalized boosted model-based propensity scores. Matching was conducted on all patients with complete data on age at diagnosis, institution, period of diagnosis, tumor size, and grade (*n* = 1303); no patients were excluded by the matching.

Separate propensity score matching analyses were performed for a 5-class treatment variable indicating whether a given patient received breast conserving surgery, breast conservation surgery with radiotherapy, breast conservation surgery with endocrine therapy, breast conservation surgery with both radiotherapy and endocrine therapy, or mastectomy, and a 7-class treatment variable further sub-setting patients who received endocrine therapy into those who received less or more than two years of treatment.

To estimate the marginal hazard ratio (HR), we fit a univariate Cox proportional hazard model with time to second breast cancer event as outcome and the treatment variable, and included the weights from propensity score matching in the estimation.

## Supplementary information

Supplementary Tables 1-6

## Data Availability

Data is available upon reasonable request. Data for analysis in this article is de-identified and dates recoded, and patients cannot be reidentified.
