# Supplementary material for: Insights from a multi-institutional registry show duration of endocrine treatment for DCIS impacts second events
Source: NPJ Breast Cancer. 2025 Jul 1;11:63. doi: 10.1038/s41523-025-00774-3 (PMC12216362; doi:10.1038/s41523-025-00774-3)
Supplement: Supplementary file 1 — Supplementary Tables 1-6 [file 41523_2025_774_MOESM1_ESM.pdf]

List of Supplementary Tables

Supplemental Table 1: Event Counts…………………………………………………………………………..Pg 2

Supplemental Table 2: Association between Second Events and Clinical Covariates……………………..Pg 4

Supplemental Table 3: Subset analysis restricting to patients with >= 2 years of follow-up who did not have a 2nd event within 2 years………………………………………………………………………………Pg 5

Supplemental Table 4: Subset analysis evaluating the association between any second event and endocrine therapy among patients who received breast-conserving surgery without radiation and with radiation…………………………………………………………………………………………………Pg 6

Supplemental Table 5: Propensity Score Weighted Cox proportional hazard models……………………..Pg 7

Supplemental Table 6: Subset Analysis Restricted to ER+ Patients…………………………………………..Pg 8

Supplemental Table 1: First Recurrent Breast Cancer Event Type by Treatment Group and Year of Recurrent Event

Year	BCS	BCS+RT	BCS+ET	BCS+ET+RT	Mastectomy	Total
**0-1**	**10**	**5**	**3**	**0**	**9**	**27**
Unknown	0	1	0	0	0	1
Ipsilateral Invasive	2	0	1	0	4	7
Ipsilateral DCIS	6	1	1	0	0	8
Contralateral/Metastatic	2	3	1	0	5	11
**1-2**	**19**	**8**	**1**	**1**	**8**	**37**
Unknown	0	0	0	0	0	0
Ipsilateral Invasive	5	2	0	0	4	11
Ipsilateral DCIS	13	5	1	0	0	19
Contralateral/Metastatic	1	1	0	1	4	7
**2-3**	**11**	**9**	**1**	**2**	**5**	**28**
Unknown	3	1	0	0	0	4
Ipsilateral Invasive	2	0	1	0	0	3
Ipsilateral DCIS	6	5	0	1	1	13
Contralateral/Metastatic	0	3	0	1	4	8
**3-4**	**8**	**4**	**2**	**1**	**2**	**17**
Unknown	0	0	0	0	0	0
Ipsilateral Invasive	2	1	0	1	1	5
Ipsilateral DCIS	3	1	2	0	0	6
Contralateral/Metastatic	3	2	0	0	1	6
**4-5**	**8**	**1**	**2**	**3**	**2**	**16**
Unknown	0	0	0	0	0	0
Ipsilateral Invasive	1	1	0	1	0	3
Ipsilateral DCIS	4	0	2	2	1	9
Contralateral/Metastatic	3	0	0	0	1	4
**5-6**	**6**	**4**	**1**	**0**	**0**	**11**
Unknown	0	0	0	0	0	0
Ipsilateral Invasive	2	2	1	0	0	5
Ipsilateral DCIS	4	2	0	0	0	6
Contralateral/Metastatic	0	0	0	0	0	0
**6-7**	**6**	**5**	**0**	**2**	**4**	**17**
Unknown	0	0	0	0	0	0
Ipsilateral Invasive	2	1	0	0	1	4
Ipsilateral DCIS	3	1	0	1	0	5
Contralateral/Metastatic	1	3	0	1	3	8
**7-8**	**1**	**0**	**1**	**0**	**6**	**8**
Unknown	0	0	0	0	0	0
Ipsilateral Invasive	0	0	0	0	0	0
Ipsilateral DCIS	0	0	0	0	0	0
Contralateral/Metastatic	1	0	1	0	6	8
**8-9**	**0**	**2**	**2**	**2**	**2**	**8**

Unknown	0	0	0	0	0	0
Ipsilateral Invasive	0	2	0	2	0	4
Ipsilateral DCIS	0	0	2	0	0	2
Contralateral/Metastatic	0	0	0	0	2	2
**9-10**	**1**	**0**	**2**	**2**	**2**	**7**
Unknown	0	0	0	0	1	1
Ipsilateral Invasive	1	0	1	0	0	2
Ipsilateral DCIS	0	0	1	0	0	1
Contralateral/Metastatic	0	0	0	2	1	3
**10-11**	**3**	**2**	**0**	**0**	**2**	**7**
Unknown	0	0	0	0	1	1
Ipsilateral Invasive	1	0	0	0	0	1
Ipsilateral DCIS	0	0	0	0	0	0
Contralateral/Metastatic	2	2	0	0	1	5
**11-12**	**0**	**0**	**1**	**0**	**1**	**2**
Unknown	0	0	0	0	0	0
Ipsilateral Invasive	0	0	0	0	0	0
Ipsilateral DCIS	0	0	1	0	0	1
Contralateral/Metastatic	0	0	0	0	1	1
**12-13**	**1**	**1**	**0**	**0**	**0**	**2**
Unknown	0	0	0	0	0	0
Ipsilateral Invasive	1	0	0	0	0	1
Ipsilateral DCIS	0	1	0	0	0	1
Contralateral/Metastatic	0	0	0	0	0	0
**13-14**	**0**	**0**	**0**	**0**	**2**	**2**
Unknown	0	0	0	0	0	0
Ipsilateral Invasive	0	0	0	0	0	0
Ipsilateral DCIS	0	0	0	0	0	0
Contralateral/Metastatic	0	0	0	0	2	2
**14-15**	**0**	**0**	**0**	**0**	**1**	**1**
Unknown	0	0	0	0	0	0
Ipsilateral Invasive	0	0	0	0	0	0
Ipsilateral DCIS	0	0	0	0	0	0
Contralateral/Metastatic	0	0	0	0	1	1
**>15***	**1**	**0**	**0**	**2**	**1**	**4**
Unknown	0	0	0	0	0	0
Ipsilateral Invasive	0	0	0	1	0	1
Ipsilateral DCIS	0	0	0	0	0	0
Contralateral/Metastatic	1	0	0	1	1	3
**Total**	**75**	**41**	**16**	**15**	**47**	**194**
Unknown	3	2	0	0	2	7
Ipsilateral Invasive	19	9	4	5	10	47
Ipsilateral DCIS	39	16	10	4	2	71
Contralateral/Metastatic	14	14	2	6	33	69

*** Second events occurring after 15 years are censored from analysis**

Supplemental Table 2: Association between Second Events and Clinical Covariates

	N	Hazard Ratio		LCI	UCI	Wald test p
**Age**						
Age < 50	637		REF			
Age >= 50	1279	0-7.5 yr	0.62	0.45	0.85	0.003
		7.5-15 yr	2.45	1.07	5.62	0.034
**Period of Diagnosis**						
1985-1993	53		1.03	0.46	2.3	0.948
1994-2001	279		1.45	0.95	2.22	0.084
2002-2009	704		1.12	0.77	1.63	0.553
2010-2017	880		REF			
**Size**						
<= 2cm	460		REF			
>2cm	963	0-7.5 yr	1.2	0.81	1.77	0.367
		7.5-15 yr	0.7	0.26	1.9	0.487
**Grade**						
Grade I/II	807		REF			
Grade III	855		1.18	0.86	1.62	0.3
**Comedonecrosis**						
Absent	534		REF			
Present	568		1.29	0.84	1.96	0.245
**ER status**						
ER Positive	1080		REF			
ER Negative	213		1.08	0.65	1.81	0.76

LCI-Lower Confidence Interval, UCI-Upper Confidence Interval

Supplemental Table 3: Subset analysis restricting to patients with >= 2 years of follow-up who did not have a 2nd event within 2 years

**5 class treatment variable**					
	N	HR	LCI	UCI	Wald p
BCS	339				REF
BCS+RT	494	0.45	0.28	0.72	0.0010
BCS+ET	140	0.62	0.33	1.17	0.1441
BCS+ET+RT	222	0.41	0.21	0.77	0.0055
Mastectomy	490	0.40	0.25	0.64	0.0001
**7-class treatment variable**					
	N	HR	LCI	UCI	Wald p
BCS	339				REF
BCS+RT	494	0.45	0.28	0.72	0.0010
BCS+ET<=2	54	1.31	0.65	2.67	0.4518
BCS+ET>2	86	0.24	0.08	0.77	0.0163
BCS+RT+ET<=2	51	0.48	0.15	1.53	0.2147
BCS+RT+ET>2	171	0.39	0.19	0.79	0.0091
Mastectomy	490	0.40	0.25	0.64	0.0001

Supplemental Table 4: Univariate subset analyses evaluating the association between any second event and endocrine therapy among patients who received breast-conserving surgery without radiation and with radiation

	**BCS without Radiation Therapy**			**BCS with Radiation Therapy**		
	N	Hazard Ratio (95% CI)	Wald test p	N	Hazard Ratio (95% CI)	Wald test p
**Endocrine therapy**						
No	401	REF		572	REF	
Any	152	0.53 (0.31 - 0.90)	0.020	252	0.68 (0.36 - 1.27)	0.224
**Endocrine therapy**						
No	401	REF		572	REF	
<= 2 years	58	0.99 (0.52 - 1.86)	0.965	64	0.96 (0.34 - 2.69)	0.942
> 2 years	94	0.26 (0.10 - 0.64)	0.004	188	0.60 (0.29 - 1.24)	0.166

Supplemental Table 5: Propensity Score Weighted Cox proportional hazard models

5 class treatment variable						
	Unadjusted Sample Size	Effective Sample Size	HR	LCI	UCI	Wald p
BCS	257	172.20				REF
BCS+RT	407	320.02	0.35	0.19	0.63	0.0006
BCS+ET	96	38.87	0.33	0.14	0.77	0.0104
BCS+ET+RT	186	135.10	0.39	0.19	0.80	0.0108
Mastectomy	357	230.21	0.33	0.19	0.57	0.0001
**7-class treatment variable**						
	Unadjusted Sample Size	Effective Sample Size	HR	LCI	UCI	Wald p
BCS	257	173.99				REF
BCS+RT	407	322.56	0.35	0.19	0.64	0.0008
BCS+ET<=2	35	13.76	0.73	0.26	2.02	0.5449
BCS+ET>2	61	36.45	0.07	0.02	0.30	0.0004
BCS+RT+ET<=2	45	26.02	0.26	0.07	0.99	0.0488
BCS+RT+ET>2	141	112.81	0.44	0.20	0.95	0.0365
Mastectomy	357	230.88	0.31	0.18	0.53	<0.0001

Supplemental Table 6: Subset analysis restricting to ER+ patients

**5 class treatment variable**					
	N	HR	LCI	UCI	Wald p
BCS	192				REF
BCS+RT	335	0.40	0.22	0.72	0.0020
BCS+ET	100	0.35	0.14	0.84	0.0195
BCS+ET+RT	196	0.31	0.15	0.64	0.0015
Mastectomy	257	0.41	0.22	0.76	0.0048
**7-class treatment variable**					
	N	HR	LCI	UCI	Wald p
BCS	192				REF
BCS+RT	335	0.40	0.22	0.72	0.0020
BCS+ET<=2	33	0.55	0.17	1.82	0.3306
BCS+ET>2	67	0.25	0.08	0.83	0.0240
BCS+RT+ET<=2	53	0.26	0.06	1.09	0.0659
BCS+RT+ET>2	143	0.33	0.15	0.71	0.0050
Mastectomy	257	0.41	0.22	0.76	0.0048
